# Contralateral Prophylactic Mastectomy Consensus Statement from the American Society of Breast Surgeons: Additional Considerations and a Framework for Shared Decision Making

**DOI:** 10.1245/s10434-016-5408-8

**Published:** 2016-07-28

**Authors:** Judy C. Boughey, Deanna J. Attai, Steven L. Chen, Hiram S. Cody, Jill R. Dietz, Sheldon M. Feldman, Caprice C. Greenberg, Rena B. Kass, Jeffrey Landercasper, Valerie Lemaine, Fiona MacNeill, Julie A. Margenthaler, David H. Song, Alicia C. Staley, Lee G. Wilke, Shawna C. Willey, Katharine A. Yao

**Affiliations:** 1 Department of Surgery, Mayo Clinic, Rochester, MN USA; 2 Department of Surgery, David Geffen School of Medicine at UCLA, UCLA Health Burbank Breast Care, Burbank, CA USA; 3 Department of Surgery, OasisMD, San Diego, CA USA; 4 Department of Surgery, Memorial Sloan Kettering Cancer Center, New York, NY USA; 5 Department of Surgery, Case Western Reserve School of Medicine, Seidman Cancer Center, Cleveland, OH USA; 6 Department of Surgery, Columbia University, New York, NY USA; 7 Department of Surgery, University of Wisconsin, Madison, WI USA; 8 Department of Surgery, College of Medicine, Pennsylvania State University, Hershey, PA USA; 9 Department of Surgery, Gundersen Health System, Lacrosse, WI USA; 10 Department of Surgery, Royal Marsden Hospital, London, UK; 11 Department of Surgery, Center for Advanced Medicine, Breast Health Center, St. Louis, MO USA; 12 Department of Surgery, University of Chicago, Chicago, IL USA; 13 Akari Healthcare, Charleston, MA USA; 14 Department of Surgery, Medstar Georgetown University Hospital, Washington, DC USA; 15 Department of Surgery, NorthShore University Health System, Evanston, IL USA

The American Society of Breast Surgeons (ASBrS) encourages an evidence-based and patient value focused approach to contralateral prophylactic mastectomy (CPM). The ASBrS convened a panel of experts to develop a consensus statement on CPM.^1^ The majority of women will obtain no oncologic benefit from CPM, and therefore CPM should be discouraged in average-risk women with unilateral breast cancer. Consideration of the patient’s preferences and values and an informed discussion of the risks and benefits of CPM is recommended for all patients pursuing mastectomy, along with a direct recommendation by the surgeon for or against CPM.

## Sentinel Lymph Node Surgery for CPM

The benefit of performing sentinel lymph node (SLN) surgery at the time of CPM is that the lymph nodes have been evaluated in the event that an occult malignancy is found, but the downside is increased surgical morbidity such as lymphedema. By meta-analysis, the risk of lymphedema after SLN alone is 5.6 % (95 % CI 6.1–7.9 %) and increases with longer follow-up.^2^ The chance of finding occult invasive disease in a prophylactic mastectomy is 1.8 %.^3^,^4^ An additional small percent of CPM specimens harbor noninvasive disease that would not require nodal evaluation. The rate of nodal positivity in patients with occult malignancy in CPM is only 1.3 %.^3^,^5^,^6^ Considering these data, routine SLN surgery at time of CPM places more patients at risk of lymphedema than would be expected from the 1–2 % of patients with occult disease undergoing axillary dissection.^7^ Therefore the consensus group does not recommend routine SLN for CPM.

Patients at higher risk of contralateral occult malignancy are postmenopausal patients, those with triple-negative, locally advanced, inflammatory, or invasive lobular disease.^3^,^8^–^10^ MRI at the time of breast cancer diagnosis identifies occult contralateral disease 2–4 % of the time.^11^ Suspicious lesions in the contralateral breast should be biopsied, but if a biopsy is not done, SLN surgery should be considered for highly suspicious lesions.

*Summary* Sentinel lymph node surgery on the CPM side should not be routinely performed.

## Cost of CPM Versus Surveillance

There is robust literature to support the use of CPM as a cost-effective strategy in patients with hereditary breast cancer syndromes.^12^–^16^ Anderson et al. demonstrated that the most cost-effective strategy, with and without quality adjustment, for women with BRCA1 or BRCA2 mutations was prophylactic bilateral salpingo-oophorectomy with bilateral mastectomy.^12^

Simulation models analyzing costs for CPM versus surveillance in patients with sporadic breast cancer reveal disparate findings.^17^,^18^ An initial Markov model study found that CPM was cost effective compared with surveillance for patients younger than 70 years, but this finding was highly dependent on the quality of life assumptions.^17^ A second study that included operative complications and breast reconstruction costs used a decision-tree model and concluded that although CPM resulted in a cost savings over surveillance for women younger than 50 years, it also reduced quality of life years.^18^ When MRI was inserted in the model as the primary method of screening, the cost-effectiveness of CPM increased. Loss of quality of life years was largely attributed to complications from reconstructive procedures. The two models differ in the assumptions regarding quality of life. If we assume an improvement in quality of life after CPM, then CPM could be cost effective. Alternatively, if quality of life is decreased, CPM would not be a cost-effective strategy. The available data on cost effectiveness for CPM is limited to modeling studies and therefore does not provide strong scientific evidence to support CPM as a cost effective strategy.

*Summary* CPM is a cost-effective strategy for women with BRCA mutations. At this time, there is insufficient evidence to support the concept of superior cost effectiveness for CPM in women with sporadic breast cancer and the cost effectiveness is highly dependent on the quality of life assumptions.

## Impact of CPM on Psychosocial Outcomes

The decision to undergo CPM is intensely personal and frequently driven by a shifting balance between perceived future breast cancer risk, anxiety over annual screening and potential future diagnostic procedures, and the unknown physical, emotional, and cosmetic outcomes of the surgery.

Long-term outcomes for women who have undergone CPM report that 86–90 % of respondents were satisfied with the decision to undergo prophylactic surgery.^19^–^21^ With 20 years of follow-up more than 90 % of women definitely or probably would choose to undergo CPM again.^22^ However, many of these same women report dissatisfaction with areas such as body image, chronic pain, problems with implants, and sexual changes even though they noted overall satisfaction with their decision making.^23^ In a study of 296 women who participated in the National Prophylactic Mastectomy Registry and provided detailed responses to a survey evaluating their outcomes with CPM, only 6 % expressed regrets with the decision; but of these women 39 % reported poor cosmetic outcomes and 22 % reported a reduced sense of sexuality.^24^ Studies with longer follow-up had outcome data only on a proportion of the initial cohort, introducing possible bias between responders and nonresponders, limiting the strength of the evidence.

Few studies have examined quality of life between CPM and non-CPM patients. One study, approximately 10 years ago, showed no difference in quality of life between patients undergoing CPM and those undergoing unilateral mastectomy or lumpectomy.^21^ In a study from Sweden, no differences in overall health-related quality of life were identified up to two years post surgery in 60 women undergoing (delayed) CPM.^25^

*Summary* While 80–90 % of women report satisfaction with their decision to undergo CPM, 20–30 % of these women report postsurgical dissatisfaction with cosmesis, body image, and sexuality. Studies show that CPM does not affect overall quality of life parameters. Women should be counseled on the potential long-term outcomes of CPM on body image and sexuality.

## Should Performance of CPM be a Quality Measure?

Quality measures are used to compare the performance of individual surgeons or institutions and can be viewed as “external” or “internal”. External quality measures can support pay-for-performance programs or be used for public reporting and are designed to help patients and purchasers make healthcare choices among providers, while internal measures are primarily used to identify quality-improvement initiatives within a given healthcare system or hospital. In evaluating the use of CPM as a quality measure, the most important consideration is how it would impact behavior of the surgeon, hospitals, purchasers, and payors and ultimately patient care. If CPM is a publicly reported measure, one could argue that patients could self-select for surgeons who align with their preferences based on reported CPM rates, but it could also pressure surgeons to decline to perform this procedure. CPM should not be used as a high-stakes quality measure such as for public reporting or selective referral because of a lack of a clearly defined outcome that is improved for all patients, ambiguity around exclusion criteria for the denominator, and significant potential for unintended consequences that can limit access to appropriate care.

The best potential for CPM is as an internal measure to inform performance review and quality-improvement efforts. Quality measures that are used for internal reporting do not require the same level of rigor and validity as those used for higher-stakes measurement. Such quality measures can be critical in determining the etiology of overutilization, particularly for a procedure such as CPM where there is wide variation in observed rates by surgeon and hospital that may represent provider bias rather than patient preference.

In summary, the use of CPM as a quality measure is limited because of a lack of a clear association with an improved outcome and its potential for unintentionally decreasing access to patients who may be at high risk for contralateral cancer. Internal measurement to inform a better understanding of the role physician and institutional bias and practice patterns could play in driving the observed increased utilization of CPM is the only potential application at this time. The ultimate goal of CPM as an internal quality measure is to minimize unnecessary, risky surgery and to track the effectiveness of new shared decision models as they become available.

*Summary* CPM should not be used as a national quality measure.

## Perspectives About Contralateral Prophylactic Mastectomy From Other Countries: The United Kingdom and Mainland Europe

The United Kingdom has significantly lower rates of CPM than the United States, although recently rates of CPM have increased in the United Kingdom with one study showing an increase from 2.0 to 3.1 %.^26^ In Switzerland, studies have not shown any increased trend for CPM.^27^ Senior surgeons on the European Breast Cancer Board (UEMS) do not feel CPM is increasing in Scandinavia, Spain, Austria, or central Europe (personal communication). CPM drivers in the United Kingdom are similar to the United States, including influence of the media, poor understanding of the risks of relapse and the limited impact of CPM on these risks, poor understanding of contralateral breast cancer risk and risks associated with breast reconstruction, and fear of recurrence. In addition, increased access to breast reconstruction, desire for symmetry, and worry about missing future cancers from mammogram screening also motivate patients to pursue CPM.^28^ A multidisciplinary approach to managing requests for CPM has been shown to reduce CPM rates.^29^

One key difference between the United Kingdom and the United States is that most private health insurers and some National Health Service commissioners in the United Kingdom will not fund CPM unless the patient is a BRCA carrier. Another key difference between the two countries is that breast surgeons in the United Kingdom learn to perform oncoplastic procedures as part of their formal training and therefore more commonly perform oncoplastic breast conservation to maintain symmetry.

UK/European Guidelines

- UK Breast Cancer clinical reference group (2016) states “There is no evidence of a survival benefit for contralateral risk-reducing mastectomy—this should not be offered, except for women with BRCA mutations, and should only be performed after a full discussion of the risks and benefits and with appropriate psychological support.”
- The National Institution for Health and Care Excellence (NICE) has no recommendations about CPM, but guidelines are due for review 2016/2017.
- EUSOMA (the European Society of Breast Cancer Specialists) and EUROPA DONNA (the European Breast Cancer Coalition) do not have any published guidelines.

*Summary* CPM rates are rising in the United Kingdom but not mainland Europe. CPM drivers are similar between the United Kingdom and the United States. Payment for CPM is not as freely available in Europe as in the United States.

## Patient Perspectives on CPM

In numerous studies, many patients arrived at a decision to have a CPM based on two main themes—a decision based in fear or a decision to “take control.” Patient’s fear translated into an “overestimated risk of recurrence, contralateral breast cancer, and death.”^30^ Breast cancer surgical treatment decisions are made when a patient’s best decision-making skills are severely impaired by the stress and anxiety of their cancer diagnosis.^31^

The increased media focus on surgical treatment options exploded after Angelina Jolie, a prominent celebrity and actress, announced her decision to undergo bilateral prophylactic mastectomy after finding out she was a BRCA1 gene mutation carrier. This increased media exposure for a woman choosing bilateral mastectomy raised the visibility of prophylactic surgical options and generated considerable confusion for other newly diagnosed patients as to whether a CPM was indicated for them. This confusion has surfaced in decision-making conversations with patients and surgeons when discussing surgical options.^32^

Ultimately, fear of cancer recurrence and input from family and friends influence decisions to undergo CPM.^33^,^34^ Although fear of recurrence is a major concern among breast cancer survivors after surgery, no standard strategies exist that qualify or alleviate this distress.^35^ Patients can regret irrevocable surgical decisions made without carefully considering all available options.^36^

*Summary* The “Jolie Effect” coupled with fear-based decision making impact patient’s consideration of CPM. Additional educational resources on risks and benefits, stronger patient engagement, and enhanced decision-making guidelines are needed.

## Shared Decision Making for CPM

Most women who have undergone CPM report having taken an active role in the decision process, with roughly 45–57 % reporting making the decision mainly on their own, and only 15–38 % reporting sharing in the decision process.^20^,^37^ Although the majority of patients who contemplate CPM identify their physicians as a key source of information, in a cohort of young women (younger than 40 years), few rate a desire to follow physician recommendation as very important in their decision making.^38^ Critical factors that were highly important to patients in the CPM decision-making process included: reducing their chance of a CBC (98 %), achieving peace of mind (95 %), improving survival/extending life (94 %), feeling at increased risk for CBC (87 %), and preventing metastatic spread (85 %).^38^

Many patients consider CPM prior to seeing their surgeon. In a recent study, more than 50 % of women with sporadic breast cancer were initially interested in CPM, but only 10 % underwent CPM.^39^ Although patients claim CPM discussion rates of 45–80 % with their doctor, only half of patients relay that their doctors outlined reasons not to have CPM.^38^,^39^ Given that approximately one-third of patients in one study experienced worse than expected results, this discussion may serve as an educational opportunity for providers to optimize informed consent.^20^ An accurate model that provides realistic numbers regarding CBC risk and impact of CPM and incorporates patient desires and other psychosocial factors may serve to enhance shared decision making between the patient and provider.

*Summary* Shared decision making that includes a comprehensive discussion of risks and benefits of CPM is important.

## Counseling Patients on Contralateral Prophylactic Mastectomy

Surgeons should strive to help patients make decisions that are informed and evidence based. They should encourage patients to make decisions that are concordant with their personal values. They should inform patients about the low risk of CBC for most patients, how CPM impacts survival and recurrence, complications and risks of CPM, and how CPM can impact body image and cosmesis. Asking patients about the importance of keeping the breast, perspectives on radiation, the importance of breast symmetry to body image, and the importance of removing the breast for peace of mind all help differentiate between those patients wanting mastectomy versus breast conservation.^40^ Surgeons should encourage patients to actively participate in the decision-making process and try to elicit patient’s treatment preferences. Since many patients consider CPM somewhere in their decision-making process, surgeons should make a recommendation based on their expert opinion after weighing the evidence and reviewing the risks and benefits of CPM with the patient.^39^ The surgeon is responsible for informing the patient about CPM’s impact on outcomes, both physical and psychological, engaging the patient in the decision-making process, and ensuring that patients are making treatment decisions that are concordant with their personal values and goals.

The consensus group has compiled a template of information that providers should include in the discussion with every patient considering CPM for unilateral breast cancer (excluding high-risk patients such as BRCA carriers) (Table 1). It is highly recommended that surgeons incorporate this template into conversations with patients to ensure that patients are making high-quality decisions about their breast surgery.

**Table 1:** CPM discussion guide—Information for patients regarding CPM. Providers should provide this information to every patient considering CPM for unilateral breast cancer (excluding high-risk patients like BRCA carriers)

For most women, the estimated risk of cancer in the opposite breast is 2–6 % over the next 10 years. This means you have a 94–98 % chance of not getting cancer in your opposite breast over the next 10 years or more.
CPM is not 100 % protective against cancer forming in your other breast.
CPM will not improve your cure rate for your known cancer.
CPM will not reduce your risk of cancer returning from your known cancer.
CPM will not reduce your need for other cancer treatments for your known cancer (adjuvant therapy), if indicated.
The risk of surgical complications at the surgical site (such as bleeding, infection, healing complications, and chronic pain) is approximately twice as high when CPM is performed.
CPM results in permanent numbness of the chest wall (and nipple if preserved).
CPM with reconstruction will result in an increased number of operations.
Complications from CPM may delay treatment of your known cancer, including chemotherapy and radiation that may be recommended after surgery.
CPM may be associated with negative impact on physical, emotional, and sexual well-being. Approximately 10 % of women regret their decision to undergo CPM.
Breast feeding will not be possible after CPM.
Women who undergo CPM will not need mammograms or routine breast imaging for cancer screening after surgery.

*Summary* CPM counseling should include discussion of CPM, risks of CPM, rates of CBC, and ensure patients are engaged in the decision making, and making decisions that are concordant with their treatment preferences and personal values.
